# The Role of Gibberellins in Regulation of Nitrogen Uptake and Physiological Traits in Maize Responding to Nitrogen Availability

**DOI:** 10.3390/ijms21051824

**Published:** 2020-03-06

**Authors:** Yubin Wang, Qingqing Yao, Yushi Zhang, Yuexia Zhang, Jiapeng Xing, Benzhou Yang, Guohua Mi, Zhaohu Li, Mingcai Zhang

**Affiliations:** 1 State Key Laboratory of Plant Physiology and Biochemistry, Engineering Research Center of Plant Growth Regulator, Ministry of Education, College of Agronomy and Biotechnology, China Agricultural University, Beijing 100193, China; wangyb_221@163.com (Y.W.); ksy18801291882@163.com (Q.Y.); zys_0909@126.com (Y.Z.); zhangyx@126.com (Y.Z.); xjp0815@163.com (J.X.); yangbz96@163.com (B.Y.); lizhaohu@cau.edu.cn (Z.L.); 2 College of Resources and Environmental Science, Department of Plant Nutrition, China Agricultural University, Beijing 100193, China; miguohua@cau.edu.cn; 3 Center for Crop Functional Genomics and Molecular Breeding, China Agricultural University, Beijing 100193, China

**Keywords:** maize, gibberellin, nitrogen, nitrate (NO_3_^−^) uptake, nitrate (NO_3_^−^) transporter

## Abstract

Modified gibberellin (GA) signaling leads to semi-dwarfism with low nitrogen (N) use efficiency (NUE) in crops. An understanding of GA-mediated N uptake is essential for the development of crops with improved NUE. The function of GA in modulating N uptake capacity and nitrate (NO_3_^−^) transporters (NRTs) was analyzed in the GA synthesis-deficient mutant z*mga3ox* grown under low (LN) and sufficient (SN) N conditions. LN significantly suppressed the production of GA_1_, GA_3,_ and GA_4_, and the *zmga3ox* plants showed more sensitivity in shoots as well as LN stress. Moreover, the higher anthocyanin accumulation and the decrease of chlorophyll content were also recorded. The net NO_3_^−^ fluxes and ^15^N content were decreased in *zmga3ox* plants under both LN and SN conditions. Exogenous GA_3_ could restore the NO_3_^−^ uptake in *zmga3ox* plants, but uniconazole repressed NO_3_^−^ uptake. Moreover, the transcript levels of *ZmNRT2.1/2.2* were downregulated in *zmga3ox* plants, while the GA_3_ application enhanced the expression level. Furthermore, the RNA-seq analyses identified several transcription factors that are involved in the GA-mediated transcriptional operation of *NRTs* related genes. These findings revealed that GAs influenced N uptake involved in the transcriptional regulation of *NRTs* and physiological responses in maize responding to nitrogen supply.

## 1. Introduction

Gibberellins (GAs) are phytohormones containing a tetracyclic diterpenoid structure that controls diverse aspects of plant growth and development in higher plants in response to environmental stimuli [1,2]. GA biosynthesis is catalyzed by a series of key enzymes, among which GA 20-oxidase (GA20ox) and GA 3-oxidase (GA3ox) are involved in the synthesis of bioactive GAs, and its deactivation is catalyzed by GA 2-oxidase (GA2ox) [1]. Mutations in GA biosynthesis genes (*GA20ox* and *GA3ox*) decrease the levels of endogenous GAs and lead to dwarfism, whereas exogenous GA treatment can restore normal growth [3,4]. DELLA proteins (DELLAs) are the major component in the GA signaling pathway. GAs promote plant growth by stimulating the degradation of the growth-repressing DELLAs [5]. Most importantly, dwarfism in rice and wheat cultivars was selected for enhancing global food production during the “green revolution”. These dwarfing characteristics were modulated by changing the GA metabolic and signaling pathways [6]. For instance, the mutant wheats *Rht-B1b* and *Rht-D1b*, encoding DELLAs, were resistant to GA-induced loss of semi-dominant GA-insensitive dwarfism [7], while the rice semidwarf−1 (*sd−1*) gene was defective in GA 20-oxidase, leading to a decline in the bioactive GA abundance [8,9], which increased the accumulation of the DELLA protein SLR1 (Slender Rice1) [10].

Previous studies have demonstrated that GA plays essential roles in many aspects of the adaptation of plant growth during the nutrient-deficiency stresses. In *Arabidopsis*, phosphate (Pi) starvation enhanced the accumulation of DELLAs in the roots, and DELLA-mediated signaling contributed to the anthocyanin accumulation [11]. Moreover, low Pi induced the expression of high-affinity Pi transporters, which are impaired in the tomato GA biosynthetic mutant *gib3* [12]. In addition, low potassium (K) promoted the accumulation of DELLA protein in the root, and the expression of high-affinity potassium transporter 5 (*AtHAK5*) was repressed in mutant *gai−1*, which reduced K uptake and decreased K deprivation tolerance [13]. Li et al. reported that a rice transcription factor (growth-regulation factor; *OsGRF4*) positively regulated N use, and an antagonistic relationship between *OsGRF4* and DELLA protein *OsSLR1* was maintained, which coordinated the plant growth and N metabolism [10]. DELLAs interact with anthocyanin pigmentation 1 (PAP1) to upregulate the expression of anthocyanin biosynthetic related genes under N deficiency [14]. However, the mechanism of N uptake and its allocation by GAs in crops subjected to different N level conditions is yet to be known.

It was reported that exogenous GA_3_ enhanced N use efficiency (NUE) in mustard, tomato, and cucumber [15,16,17]. In contrast, a decrease in N uptake was observed in mutant *sd1* and *Rht* alleles [10]. Thus, *sd1* reduced the rate of ammonium (NH_4_^+^) uptake and modulated N-responsive regulation in rice. A similar phenomenon was observed during the nitrate (NO_3_^−^) uptake in mutant wheat *Rht-B1b* plants [10]. Consequently, the dwarfism was the reason for more N fertilizer to be applied for higher yield during the “green revolution”, which resulted in lower NUE in crop production [6,18]. Plant NUE is inherently complex, and it is important to understand the function and regulation of the key components involved in N uptake, translocation, assimilation, and remobilization [19]. In consequence, GA signaling regulates the N uptake in crops, and it is necessary to enhance NUE for the development of new GA-insensitive dwarfing varieties [20]. Accordingly, it is essential to explore the physiological and molecular aspects of GAs in regulating N uptake and allocation in plants.

Maize is an important crop worldwide that is cultivated in aerobic soils. It requires a high amount of N fertilizer [21], and NUE has been the major limitation in the past 50 years [22]. It is essential to verify the physiological and molecular mechanisms in maize for breeding the N-efficient cultivars [23]. In general, nitrate is the major form of N source in aerobic soils [24], and NO_3_^−^ transporters (NRTs) assist in NO_3_^−^ uptake and its utilization throughout the lifecycle of plants [25,26]. Moreover, plant NO_3_^−^ uptake generally involves low-affinity transport systems (LATS) and high-affinity transport systems (HATS). LATS and HATS have been associated with the NPF/NRT1 and NRT2 families (including NRT2.1 and NRT2.2), respectively [25]. Moreover, the expression of primary NPF (nitrate transporter1/peptide transporter family) genes, *NPF6.3* and *NRT2.1*, can be regulated by auxin, ethylene, and cytokinin, which influence N uptake [27]. Low N reduces GA synthesis and represses plant growth. Exogenous GA_3_ can restore plant growth in maize even when subjected to low N supply [28]. Although modulating the action of GA can alter N uptake and allocation, little is known about how GA regulates N uptake in crops.

The aim of the present research is to investigate the role of GAs in the regulation of physiological responses to N supply in maize. The GA synthesis deficient mutant z*mga3ox* was constructed and combined with exogenous GA_3_ and uniconazole (an inhibitor of GA biosynthesis, Ucz) for analyzing N uptake under LN and SN conditions. Moreover, GA-mediated NO_3_^−^ uptake was evaluated by using the non-invasive micro-test (NMT) and the ^15^N labeling technique. Furthermore, an RNA-seq assay was conducted to investigate the role of GAs in modulating N uptake at the transcript profiles. Thereby, the present study demonstrates the role of the GA in regulating the physiological responses in maize in correspondence to N supply.

## 2. Results

### 2.1. Characterization of zmga3ox Mutant in Maize

**Figure 1:**
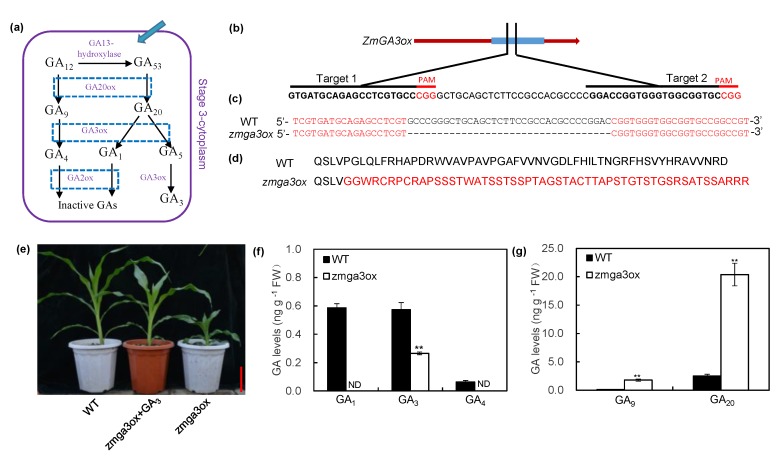
The *ZmGA3ox* knockout mutant characterization and phenotype analysis under sufficient nitrogen (SN) condition. (**a**) Schematic representations of the GAs biosynthesis pathway; (**b**) targeted mutagenesis of *ZmGA3ox* via CRISPR-Cas9; protospacer adjacent motif (PAM) sequences labelled in red; (**c**) sequence information of the gene-editing region in wild-type and *zmga3ox* plants; (**d**) alignment of the amino acid sequences of *ZmGA3ox* and *zmga3ox*. Only the sequences flanking the mutations were shown, and the frameshifted sequences in *zmga3ox* highlighted in red. (**e**) The phenotype of the *zmga3ox* and GA_3_-treated *zmga3ox* seedling. Bar = 20 cm. (**f**) The contents of GA_1_, GA_3_, and GA_4_ in shoots of *zmga3ox* and wild-type seedlings. ND: not detected. (**g**) The contents of GA_9_ and GA_20_ in shoots of *zmga3ox* and wild-type seedlings. Values were the means ± SD (*n* = 3). The asterisks indicated significant difference between wild-type and *zmga3ox* plants, as evaluated by Student’s *t*-tests ** *p* < 0.01.

The GA3ox enzymes participate in the synthesis of bioactive GAs and catalyze the conversion of GA_9_ to bioactive GA_4_ and GA_20_ to GA_1_ and GA_3_ (Figure 1a). In order to verify the role of GAs in response to N supply in maize, the knockout mutants were generated using a type II CRISPR-Cas9 system [29]. A knockout mutant named *zmga3ox* was obtained, which conferred a 34-bp deletion causing a frameshift in *ZmGA3ox* (Figure 1b–d). Compared to the wild-type plant, the expression level of *ZmGA3ox* was hardly detected in the shoots and roots of *zmga3ox* plant (Figure S1). Meanwhile, the *zmga3ox* plant displayed growth inhibition, and exogenous GA_3_ could restore normal growth (Figure 1e). The concentrations of GA_1_, GA_3_, and GA_4_ in *zmga3ox* plant were significantly lower than those in wild-type plants (Figure 1f). Also, the *zmga3ox* plant had greater levels of GA_9_ and GA_20_ compared to the wild-type (Figure 1g). These results suggest that *zmga3ox* is an endogenous GA-defective mutant.

### 2.2. GAs Altered the Anthocyanin Accumulation and Chlorophyll Content in Leaves in Response to NO_3_^−^ Supply

**Figure 2:**
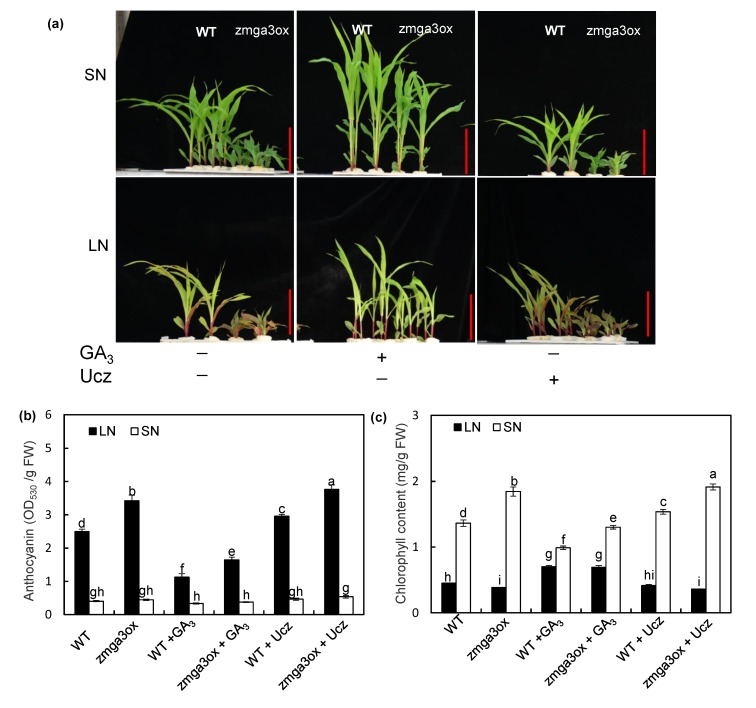
The anthocyanin accumulation and chlorophyll content in *zmga3ox* leaves under LN or SN condition. (**a**) Appearance of the GA_3_ or Ucz-treated wild-type and *zmga3ox* leaves at 7 d after LN or SN treatment. Bar = 10 cm. (**b**) The content of anthocyanin in *zmga3ox* shoots treated with GA_3_ or Ucz at 7 d after LN or SN treatment. Values were the means ± SD (*n* = 3). (**c**) The content of chlorophyll in *zmga3ox* shoots treated with GA_3_ or Ucz at 7 d after LN or SN treatment. Values were the means ± SD (*n* = 3). Different letters indicated significant difference calculated by Fisher’s LSD (*p* < 0.05).

The anthocyanin and chlorophyll contents were determined in wild-type and *zmga3ox* seedlings by growing in LN and SN solutions with or without GA_3_ and Ucz. The z*mga3ox* plants were more sensitive to LN supply than the wild-type plant, and also the z*mga3ox* leaves were more purple. As expected, the GA_3_-treated leaves were less purple, but Ucz strengthened such effect. Moreover, the application of GA_3_ and Ucz had no effect on the anthocyanin accumulation under the SN condition (Figure 2a). LN significantly enhanced the accumulation of the anthocyanin in z*mga3ox* leaves, and the anthocyanin content was increased by 3-fold under the LN condition (Figure 2b). In addition, the chlorophyll content was decreased in *zmga3ox* leaves compared to the wild-type leaves under LN condition, and exogenous GA_3_ increased the chlorophyll content in both wild-type and *zmga3ox* leaves; however, Ucz treatment slightly decreased the chlorophyll content in both wild-type and *zmga3ox* leaves (Figure 2c). GA_3_ treatment declined the chlorophyll content in *zmga3ox* leaves under SN condition, while Ucz treatment enhanced the chlorophyll content. In addition, the photosynthetic rate, leaf areas, and O_2_^−^ production rate were also measured in the wild-type and *zmga3ox* leaves treated with or without GA_3_ application. The *zmga3ox* leaves showed higher the photosynthetic rate than the wild-type leaves, while the photosynthetic rate was decreased by GA_3_ treatment (Figure S2a). Moreover, the leaf areas of the *zmga3ox* plant*s* were significantly less than those of the wild-type plants (Figure S2b). Furthermore, the O_2_^−^ production rate in *zmga3ox* leaves showed slightly lower than that in wild-type leaves (Figure S2c).

### 2.3. Supply of NO_3_^−^ Modulated the Transcript Expression of GA Biosynthesis- and Metabolism-Related Genes

**Figure 3:**
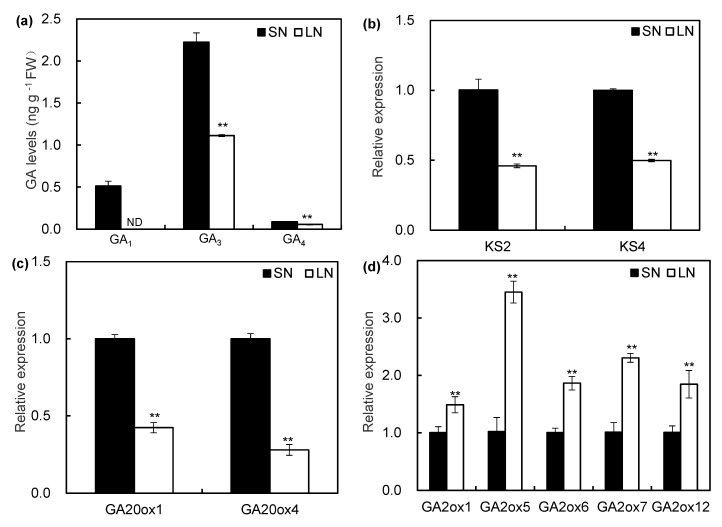
The GA contents and the expression levels of GA biosynthesis- and catabolic related genes in maize seedlings responded to LN and SN. (**a**) The contents of GA_1_, GA_3,_ and GA_4_ in the wild-type roots at 5 d after LN and SN. ND: not detected. (**b**–**d**) The transcriptional levels of ent-kaurene synthases (**b**), GA 20-oxidases (**c**), and GA 2-oxidase (**d**) genes in the wild-type roots at 3 d after LN and SN treatment. (**a**–**d**) Values were the means ± SD (*n* = 3). The asterisks indicated significant difference compared with the control as evaluated by Student’s *t*-tests ** *p* < 0.01.

In order to understand the role of GAs in maize corresponding to NO_3_^−^ supply, the content of GA_1_, GA_3,_ and GA_4_ was determined by using the HPLC–MS/MS method. LN significantly decreased the content of GA_1_, GA_3,_ and GA_4_ in roots as compared to SN (Figure 3a). The GA_1_ content could not be detected in the roots under the LN condition, while the GA_3_ and GA_4_ levels were lower under LN condition than those under the SN condition. The expression levels of *ent*-kaurene synthase genes *ZmKS2* and *ZmKS4* in GA biosynthesis pathway were significantly downregulated by LN in comparison to SN (Figure 3b). Similarly, LN significantly decreased the expression levels of *ZmGA20ox1* and *ZmGA20ox4* (Figure 3c). LN enhanced the expression levels of catabolic genes *ZmGA2ox1, ZmGA2ox5, ZmGA2ox6, ZmGA2ox7,* and *ZmGA2ox12* (Figure 3d). The GA_3_ contents, as well as the expression of genes encoding enzymes of GA metabolism, were also determined in the *zmga3ox* mutant. As shown in Figure S3, the levels of GA_3_ were less under LN condition than those under the SN condition (Figure S3a). LN significantly down-regulated the expression levels of GA biosynthetic enzyme genes *ZmKS2*, *ZmKS4, ZmGA20ox1,* and *ZmGA20ox4* (Figure S3b,c). Inversely, LN enhanced the expression levels of catabolic enzyme genes *ZmGA2ox1, ZmGA2ox5, ZmGA2ox6, ZmGA2ox7,* and *ZmGA2ox12* (Figure S3d). The results indicated that LN caused a reduction in the bioactive GA_S_ levels in roots by regulating the transcripts encoding GA 20-oxidases and GA 2-oxidases.

### 2.4. GAs Involved in Manipulating the NO_3_^−^ Uptake

**Figure 4:**
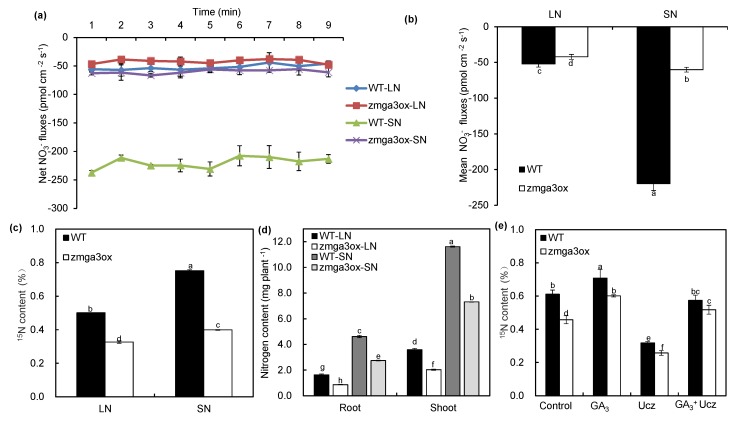
Nitrate uptake and allocation in *zmga3ox* plants under LN and SN conditions. (**a**–**b**) The net NO_3_^−^ flux (**a**), and the mean NO_3_^−^ flux (**b**) along the maturation zone of wild-type and *zmga3ox* primary root. Values were the means ± SD (*n* = 7). (**c**) The ^15^N content after 10 min ^15^N tracing assay in wild-type and *zmga3ox* plants; (**d**) total N content per plant in shoots and roots of wild-type and *zmga3ox* plants; (**e**) the ^15^N content in wild-type and *zmga3ox* seedlings treated with GA_3_ and/or uniconazole (Ucz). (**c**–**e**) Values were the means ± SD (*n* = 3). Different letters indicated significant difference calculated by Fisher’s LSD (*p* < 0.05).

In order to characterize the function of GAs in regulating the N uptake, the net NO_3_^−^ fluxes were measured by using the NMT technique in wild-type and *zmga3ox* roots. As shown in Figure 4a, the uptake of NO_3_^−^ was evidently decreased in *zmga3ox* root compared to wild-type under SN and LN conditions. The mean NO_3_^−^ fluxes were lower by 266% and 23.8% in *zmga3ox* root than wild-type under SN and LN conditions, respectively (Figure 4b). Moreover, LN repressed the ^15^N content in both wild-type and *zmga3ox* roots. The amount of ^15^N in *zmga3ox* roots was less than that in the wild-type roots under LN and SN conditions (Figure 4c). LN reduced the N content in root and shoot, and the *zmga3ox* plants had less N content than the wild-type plants under LN and SN conditions (Figure 4d). The N contents in *zmga3ox* root and shoot were less by 89.5% and 77.2% than those in wild-type under LN condition, and similar results were recorded for SN.

In order to further investigate the role of GAs in the regulation of N uptake, exogenous GA_3_ and Ucz were applied. GA_3_ treatment could increase the amount of ^15^N in wild-type and *zmga3ox* plants (Figure 4e). On the other hand, Ucz treatment significantly decreased the amount of ^15^N in wild-type and *zmga3ox* plants. The combined GA_3_ and Ucz treatments restored the amount of ^15^N in Ucz-treated plants while decreased that in GA_3_-treated plants.

To clarify the effects of GA on N uptake whether derived from differences in plant growth and nutritional status, the dry weight and total N content in the wild-type and *zmga3ox* plants were analyzed under LN or SN condition. Although the dry weight of shoots and roots were lower in *zmga3ox* plants than the wild-type plants under both LN and SN conditions, the inhibition rate of LN to SN in dry biomass of shoot or root presented no significant difference between wild-type and *zmga3ox* plants (Figure S4a–d). Similarly, the *zmga3ox* plants had lower total N content in shoots and roots compared to the wild-type plants. However, the inhibition rate of LN to SN in total N content of shoot or root showed significant difference between wild-type and *zmga3ox* plants (Figure S4e–h).

### 2.5. GAs Modulated the Transcript Expression of NO_3_^−^ Uptake-Related Genes

**Figure 5:**
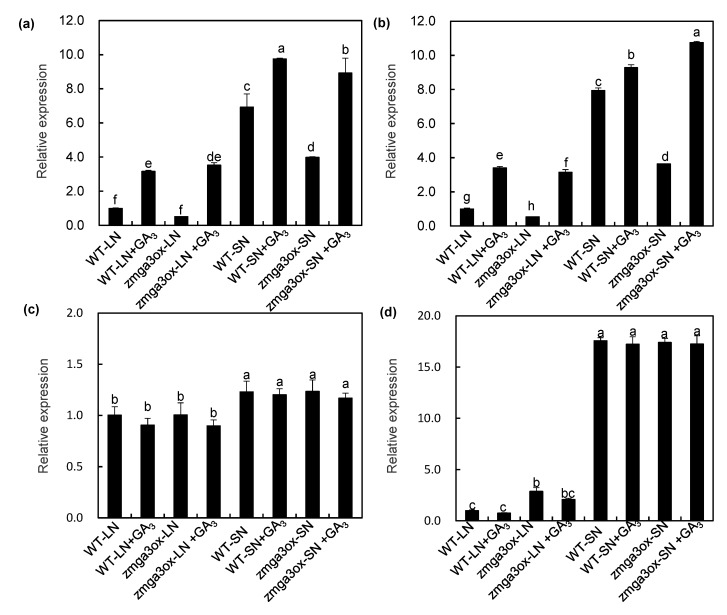
Effects of exogenous GA_3_ on the expression levels of *ZmNRT2.1* (**a**), *ZmNRT2.2* (**b**), *ZmNPF6.3a* (**c**)*,* and *ZmNPF6.3b* (**d**) in wild-type and *zmga3ox* roots under LN and SN conditions. The roots were harvested at 72 h after GA_3_ treatment under LN and SN conditions. Values were the means ± SD (*n* = 3). Different letters indicated significant difference calculated by Fishe’s LSD (*p* < 0.05).

In order to ascertain the mechanism of GA affecting the N uptake, the expression levels of NO_3_^−^ uptake-related genes were detected in wild-type and *zmga3ox* roots subjected to SN and LN. As shown in Figure 5, the expression levels of *ZmNRT2.1* and *ZmNRT2.2* in *zmga3ox* roots were lower than those of wild-type roots under LN and SN conditions (Figure 5a,b). The transcript expression of *ZmNRTs* was also detected in the GA_3_-treated wild-type and *zmga3ox* plants. GA_3_ treatment could significantly enhance the expression levels of *ZmNRT2.1* and *ZmNRT2.2* in wild-type and *zmga3ox* plants under LN and SN conditions. Meanwhile, LN downregulated the expression of *ZmNPF6.3b* in wild-type and *zmga3ox* roots, while the expression level of *ZmNPF6.3a* showed no significant difference between wild-type and *zmga3ox* roots (Figure 5c). The *zmga3ox* root had higher expression levels of *ZmNPF6.3b* than the wild-type root under the LN condition (Figure 5d). In addition, the expression levels of *ZmNPF6.3a* and *ZmNPF6.3b* showed no significant difference in GA_3_-treated both wild-type and *zmga3ox* plants under both LN and SN conditions.

### 2.6. RNA-Sequencing Revealed Differentially Expressed Genes (DEGs) in the Wild-Type and zmga3ox Plants in Response to NO_3_^−^ Supply

**Figure 6:**
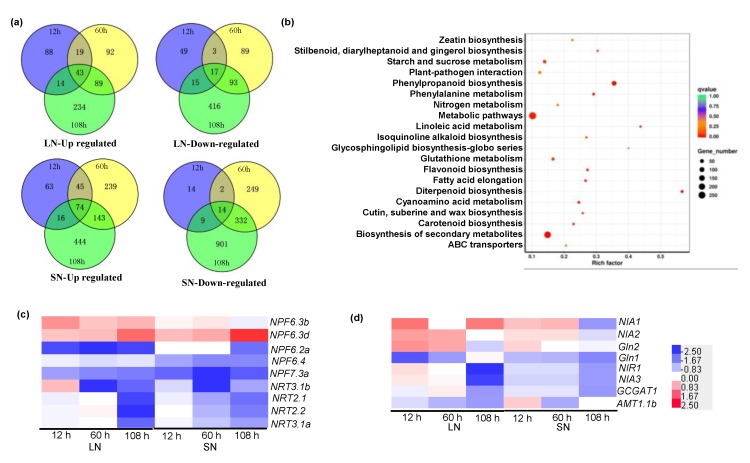
The DEGs involved in NO_3_^−^ uptake and metabolism. (**a**) Venn diagrams showed the up- and downregulated significantly differentially expressed genes (DEGs) identified by RNA-seq in the wild-type and *zmga3ox* roots at 12, 60, and 108 h after LN and SN treatments, respectively; (**b**) list of the top 20 significant enriched KEGG pathway of significantly regulated DEGs. (**c**,**d**) Heatmap showed the DEGs involved in NO_3_^−^ uptake and transport (**c**), N assimilation and NH_4_^+^ uptake (**d**). Genes annotated by MaizeGDB gene model were shown with gene name. Different expression levels were shown as log_2_FC, blue for downregulation and red for upregulation, as shown in color bar.

In order to gain molecular insights into the roles of GAs in the regulation of N uptake, the RNA-seq assay was conducted. This helped identify the differentially expressed genes (DEGs) in the wild-type and *zmga3ox* roots at 12, 60, and 108 h after the LN or SN treatment. The statistics of the clean reads in the RNA-seq are shown in Table S1. A total of 3054 DEGs were identified after filtering with a threshold of |log_2_FC| ≥ 1 under the FDR (false discovery rate) <0.05 (Table S2). The Venn diagrams showed the number of DEGs in different samples. A greater number of DEGs were downregulated compared to the number of upregulated DEGs under both the LN and SN conditions. Furthermore, a greater number of DEGs were identified under the SN treatment compared to the LN treatment. The samples collected after 108 h showed the maximum number of DEGs under the LN as well as SN conditions (Figure 6a). Besides, the heat map suggested that the level of change (fold change) in the expression of these DEGs was higher after 60 and 108 h than that after the 12 h treatment (Figure S5).

The GO (Gene Ontology) database and KEGG (Kyoto Encyclopedia of Genes and Genomes) enrichment analysis were employed to investigate the function of the 3054 DEGs and to understand the regulatory pathways that respond to the N supply in the *zmga3ox* mutant (Tables S3 and S4). The biosynthesis of phenylpropanoid and secondary metabolites was highly enriched in these DEGs (Figure 6b and Table S4). Moreover, the KEGG analysis revealed that the most significantly enriched pathway was the N-metabolism pathway, and this included the genes related to the NO_3_^−^ transporter, NO_3_^−^ reductase, and glutamate synthase (Figure 6b and Table S4). Thus, three NPF/NRT1 genes were downregulated, including *ZmNPF6.2a, ZmNPF6.4,* and *ZmNPF7.3a.* On the contrary, the expression of *ZmNPF6.3b* was upregulated under LN condition, and the expression of *ZmNPF6.3d* was upregulated under both LN as well as SN conditions (Figure 6c and Table S5). Also, two *ZmNRT2s* genes were identified and these *ZmNRT2.1* and *ZmNRT2.2* genes were downregulated. Moreover, the *ZmNRT3.1a* and *ZmNRT3.1b* genes also showed downregulated expression (Figure 6c and Table S5). The other eight important DEGs were identified as the N assimilation-related genes, including *ZmNIA1*, *ZmNIA2, ZmGln2, ZmGln1, ZmNIR1, ZmNIA3, ZmGOGAT1* and the NH_4_^+^ transporter gene *ZmAMT1.1b* (Figure 6d and Table S5); most of these important DEGs were downregulated in the *zmga3ox* plants.

**Figure 7:**
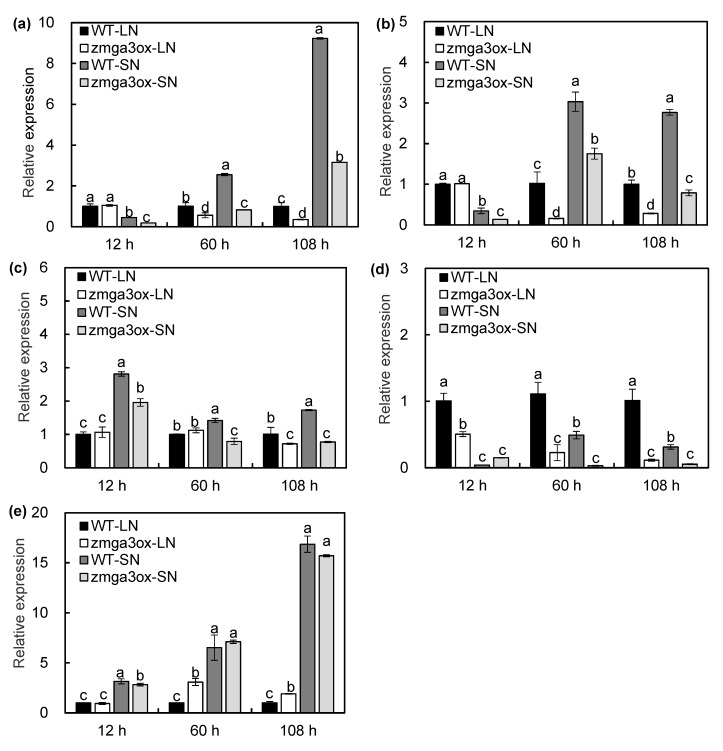
The expression levels of *ZmNRT2.1* (**a**), *ZmNRT2.2* (**b**), *ZmNPF6.4* (**c**), *ZmNPF7.3a* (**d**), and *ZmNPF6.3b* (**e**) in wild-type and *zmga3ox* roots under LN and SN conditions. Roots were harvested at 12, 60, and 108 h after LN and SN treatments. Values were the means ± SD (n = 3). Different letters indicated significant difference between the wild-type and *zmga3ox* plants at the same time point calculated by Fisher’s LSD (*p* < 0.05).

In order to further investigate the role of GAs in the transcript regulation of N uptake and assimilation, the transcripts of the important DEGs involved in this process were evaluated by the RT-qPCR analysis. As expected, all the selected genes had similar expression patterns in the RNA-seq results (Figure 7 and Table S5). LN decreased the expression levels of *ZmNRT2.1* and *ZmNRT2.2* compared to SN at 60 and 108 h after treatment, and the expression levels of *ZmNRT2.1* and *ZmNRT2.2* in the *zmga3ox* plant were lower than those in the wild-type plant, except for 12 h after LN treatment (Figure 7a,b). Similar trends were observed in the expression of *ZmNPF6.4* under SN condition (Figure 7c). However, the expression levels of *ZmNPF7.3a* were upregulated by LN compared to that by SN, and the *zmga3ox* plant showed lower expression levels of *ZmNPF7.3a* than the wild-type plant after 60 and 108 h of the LN or SN treatments (Figure 7d). Inversely, LN significantly downregulated the expression levels of *ZmNPF6.3b* compared to SN, and the expression levels of *ZmNPF6.3b* in the *zmga3ox* plant were higher than those in the wild-type plant after 60 and 108 h of the LN treatment (Figure 7e). Meanwhile, the LN treatment downregulated the expression levels of *ZmGOGAT1* and *ZmNIR1* compared to the SN treatment, and the expression levels of *ZmGOGAT1 and ZmNIR1* in the *zmga3ox* plant were lower than those in the wild-type plant at 60 and 108 h after the LN and SN treatments (Figure S6a,b). Similarly, the expression of *ZmGln1* and *ZmNIA1* was repressed at 60 and 108 h after the LN treatment, while the *zmga3ox* plant showed downregulated trend of *ZmGln1* and *ZmNIA1* than the wild-type plant (Figure S6c,d). Correspondingly, LN reduced the activities of nitrate reductase (NR) and glutamine synthase (GS); the activities of NR and GS in the *zmga3ox* plant were significantly lower than those in the wild-type plant (Figure S7).

### 2.7. Transcription Factors Involved in GA-Mediated NO_3_^−^ Uptake

**Figure 8:**
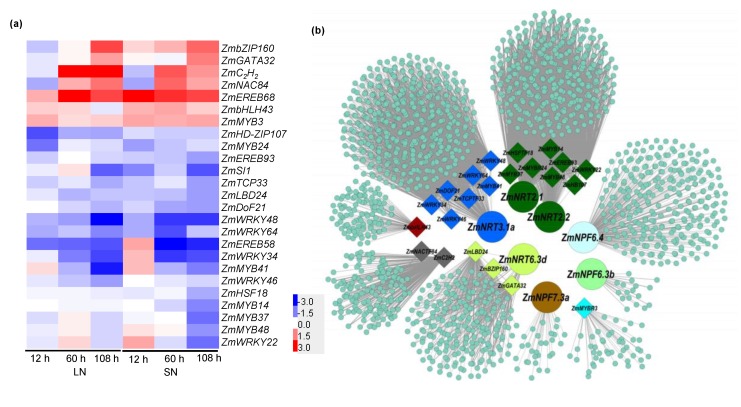
GAs regulated the expression of transcription factors in maize responded to NO_3_^−^ supply. (**a**) Heatmap showing the significant expression of transcription factors in the 2-kb putative promoter region of nitrate transporter genes. Different expression levels were shown as log_2_FC, blue for downregulation and red for upregulation, as shown in the color bar. (**b**) Weighted gene co-expression network analysis (WGCNA) of DEGs.

The transcriptional control of NO_3_^−^ uptake is well-documented over the past decade, in which transcription factors (TFs) play an important regulatory role [30]. The RNA-seq analysis revealed that a total of 287 important DEGs were identified as TFs (Table S6). To predict the targets of transcriptional regulation, the TF-binding sites were predicted from the putative promoter sequences (2 kb upstream from the transcriptional start site) of the nine identified nitrate transporter genes. As shown in Figure 8a and Table S7, 25 TFs were predicted to be involved in the regulation of nitrate transporter genes. The number of binding sites of the *MYB*, *WRKY*, and *ERF* families was the greatest, followed by the *LBD*, *TCP,* and *bZIP* family TFs.

**Figure 9:**
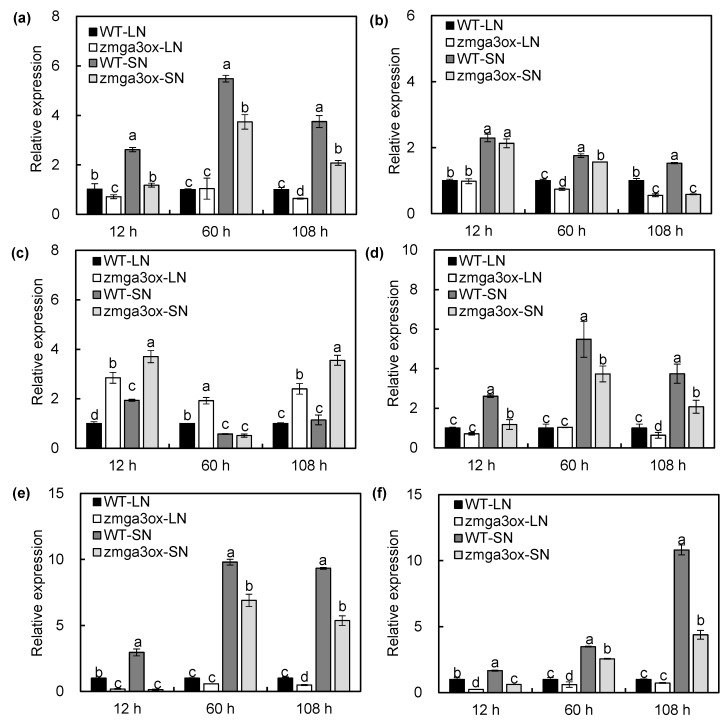
The expression levels of *ZmTCP33* (**a**), *ZmLBD24* (**b**), *ZmBbZIP160* (**c**), *ZmWRKY34* (**d**), *ZmEREB93* (**e**), and *ZmMYB41* *(***f**) in wild-type and *zmga3ox* roots under LN and SN conditions. Roots were harvested at 12, 60, and 108 h after LN and SN treatments. Values were the means ± SD (*n* = 3). Different letters indicated significant difference between the wild-type and *zmga3ox* plants at the same time point calculated by Fisher’s LSD (*p* < 0.05).

In order to investigate the potential correlations between the DEGs, a weighted-genes co-expression network analysis (WGCNA) was performed with the 3054 DEGs. As shown in Figure 8b, the genes related to nitrate transport were clustered along with the TFs displaying a predicted potential interaction into different colored modules; the genes with the same color suggest a strong co-expression relationship. For instance, *ZmNRT2.1* and *ZmNRT2.2* strongly co-expressed with *ZmMYB14/24/37/48*, *ZmEREB93*, *ZmWRKY22*, *ZmHSF18*, *and ZmHB107.* Thereafter, *ZmTCP33*, *ZmLBD24*, *ZmbZIP160*, *ZmWRKY34*, *ZmERF98*, and *ZmMYB41* were selected for the RT–qPCR assay. The expression patterns of these genes showed a similar trend with the RNA-seq data. On the contrary, the expression levels of these TFs showed a downregulated trend in the *zmga3ox* plants under the LN and SN conditions, except for *ZmbZIP160*, which showed a marked upregulation (Figure 9). These results suggest that the GA-mediated N-uptake could occur by the transcriptional regulation of the expression of the *ZmNRTs.*

## 3. Discussion

The manipulation of the GA biosynthetic and signaling genes can modulate the plant stature and lead to a “green revolution” in the cereal crops [7,8]. However, the dwarfism contributed by the GA-related mutants occurs a lower NUE in rice and wheat production [6,18]. Actually, the mutant *sd1* rice plants show a lower NH_4_^+^ uptake rate than the wild-type plants, and a lower ^15^NO_3_^−^ uptake is also observed in the mutant *Rht-B1b* wheat plants [10]. In the present study, a mutant maize *zmga3ox* was constructed and these plants showed the dwarfed phenotype, while the addition of exogenous GA_3_ could restore normal growth (Figure 1). Moreover, the mutant *zmga3ox* plants presented significantly lower NO_3_^−^ fluxes and ^15^NO_3_^−^ uptake, which could be restored by exogenous GA_3_ (Figure 4). These results suggest that the N uptake was decreased by the GA deficiency in maize plant, and GA accumulation might affect the maize plant for the adaptation to the N supply in the soil. Although the *zmga3ox* plants had less dry weight of shoots and roots than the wild-type plants, GA deficiency could not change the inhibition rate of LN to SN in the plant growth compared to the wild-type plants (Figure S4a–d). However, the inhibition rate of LN to SN in the N content was significantly modulated by GA deficiency (Figure S4e–h). These indicate that GA deficiency could not change the effects of NO_3_^−^-mediated plant growth, while it might have a significant effect on N uptake in maize exposed to LN or SN conditions.

Anthocyanin accumulation is a typical characteristic of N starvation, and GAs regulate the N deficiency-induced anthocyanin accumulation in *Arabidopsis* or tomato [11,12,14]. Meanwhile, low anthocyanin content in leaves is observed under normal N or Pi condition, and GA_3_ application exhibits little effects on anthocyanin accumulation. Similar results were observed in this study, where LN significantly induced anthocyanin accumulation in the *zmga3ox* plants, and this phenomenon could be weakened by GA_3_ treatment (Figure 2). Furthermore, GA deficiency had a slight effect on anthocyanin accumulation under SN condition. These results suggest that GA signals are involved in altering the LN-induced anthocyanin accumulation in maize. Meanwhile, the z*mga3ox* leaves showed higher the photosynthetic rate and chlorophyll concentration than the wild-type leaves (Figure 2c and Figure S2a); however, the leaf areas of the z*mga3ox* plant were significantly less than those of the wild-type plant (Figure S2b), which mainly caused low plant growth in the z*mga3ox* plant (Figure S4a). Contrary to the anthocyanin accumulation, LN significantly decreased the chlorophyll content and N content in the *zmga3ox* plants. Thus, the *zmga3ox* plants presented a significant difference in N content compared to the wild-type plants (Figure S4). Combined with these results, it was possible that NO_3_^−^ supply modulated the N accumulation for affecting the anthocyanin accumulation and chlorophyll content in the *zmga3ox* leaves. In addition, the anthocyanin accumulation is closely associated with the activities of the antioxidant enzyme system in tobacco [31], and higher anthocyanin concentration can decrease ROS accumulation in potato leaves [32]. Here, the O_2_^−^ content in *zmga3ox* leaves slightly lower than that in wild-type leaves, while it showed a little higher than the anthocyanin content under SN condition (Figure S2c), which suggests that the anthocyanin accumulation might be involved in affecting the ROS accumulation in *zmga3ox* leaves. Further studies could research whether anthocyanin accumulation is involved in altering photosynthesis and ROS balance in *zmga3ox* leaves.

The NO_3_^−^ is the main form of available inorganic N that is utilized by most plants from the aerobic soils, taken up by the roots and transported to the shoots before assimilation; this process is mediated by the NO_3_^−^ transporters [19]. To date, many *NPF*/*NRT* families in *Arabidopsis* and rice have been functionally identified for their involvement in the root NO_3_^−^ uptake [33]. Moreover, the expression of *AtNPF6.3* and *AtNRT2.1* is regulated by auxin, ethylene, and cytokinin in plants in response to N supply [27]. However, the process by which the GAs regulate the expression of *NRTs* in plants combined with sufficient or insufficient N is still unclear. In maize, only *ZmNPF6.3a* and *ZmNPF6.3b* genes have been cloned, *ZmNPF6.3b* is a dual-affinity nitrate transporter, while *ZmNPF6.3a* displays a low-affinity nitrate transport activity [34]. In the present study, the expression of *ZmNPF6.3a* was not significantly different in the wild-type and *zmga3ox* roots. Correspondingly, GA_3_ treatment could not alter the expression levels of *ZmNPF6.3a* and *ZmNPF6.3b* in wild-type and *zmga3ox* plants (Figure 5). These indicate that GAs might have a little effect on the transcriptional regulation of *ZmNPF6.3a* and *ZmNPF6.3b* in maize responding to NO_3_^−^ supply. Several studies have demonstrated that the *ZmNRT6.3a* and *OsNRT1.1a* display NO_3_^−^ transport activity in maize and rice, and overexpression of *OsNRT1.1a* in rice greatly improved N uptake, but the expression levels of *ZmNRT6.3a* and *OsNRT1.1a* are not regulated by N supply [34,35]. Meanwhile, the post-translational modifications of *NRT1s*, such as phosphorylation and ubiquitination, are also essential for NO_3_^−^ uptake or transport [36,37]. Here, the expression levels of *ZmNPF6.3a* and *ZmNPF6.3b* in the *zmga3ox* plant had no significant difference with those of the wild-type plants under SN condition (Figure 5c,d), but the *zmga3ox* plant had lower the N content compared to the wild-type plants (Figure 4c). These indicate whether GA could be involved in regulating the post-translational modifications of ZmNRT6.3s for altering the LATS-mediated NO_3_^−^ uptake, which would be further studied.

The NRT2.1 is the major HATS-type gene involved in the root NO_3_^−^ uptake in *Arabidopsis* [25,26]. The *AtNRT2.1*/*NRT2.2* double-knockout mutations result in up to 80% loss of the NO_3_^−^-inducible HATS (iHATS) activity and show severe growth restriction in the absence of the sole NO_3_^−^ source [38]. Both the constitutive HATS (cHATS) and iHATS activities were impaired under the LN condition in the knock-outs of *NRT2.1* in cucumber [39]. In the present study, the *zmga3ox* roots showed lower expression levels of *ZmNRT2.1* and *ZmNRT2.2*, and this phenomenon was also found by RNA-seq analysis, while the exogenous GA_3_ could upregulate the expression of these genes. Moreover, the *zmga3ox* roots had lower expression levels of *ZmNRT3.1a* and *ZmNRT3.1b*, and the functionality of NRT2.1 relies on its interacting protein NRT3.1/NAR2.1, as observed in the studies on the rice plant [40]. Thus, the NO_3_^−^ fluxes and ^15^NO_3_^−^ uptake in *zmga3ox* plants were lower than those in the wild-type plants, and the GA_3_ treatment could increase the amount of ^15^N. Together, GA deficiency affected N uptake involved in the transcriptional regulation of *ZmNRT2s* in the maize plant subjected to NO_3_^−^ supply.

According to Garnett et al., the transcript abundance of putative *ZmNRT2.1* and *ZmNRT2.2* is correlated with the root NO_3_^−^ uptake capacity in maize [41]. A similar result was also observed in the rice plant wherein the expression abundance of *OsNRT2.3a* and *OsNPF2.4* was significantly decreased in the *sd1* mutant plant under normal N condition [10]. However, high NO_3_^−^ supply increases the *NRT2* transcript levels in *Glycine max* plants, although HATS-mediated NO_3_^−^ influx presents a low level [42]. Similarly, the *HvNRT2* transcript is upregulated by 20% to 30% in the barley plants exposed to 10 mM NO_3_^−^ with tungstate (an inhibitor of nitrate reductase), while the NO_3_^−^ influx is decreased by 50% [43]. In addition, the phosphorylation is crucial for *NRT2* expression abundance in response to NO_3_^−^ supply [44,45]. These indicate that the post-transcriptional modifications on iHATS-mediated NO_3_^−^ might be exerted in the plants exposed to NO_3_^−^ supply. In addition, the expression level of NRTs is not only regulated by the NO_3_^−^ but also by the N metabolites, such as NH_4_^+^ or amino acids [40,44,46]. In addition to N metabolites, photosynthate (e.g., sucrose) may also influence *NRTs* expression [47]. Here, the KEGG analysis revealed that the most significantly enriched pathway included the N-metabolism and sucrose metabolism pathways (Figure 6b). These indicate that GAs significantly affect the N-metabolism and sucrose metabolism pathways in maize plants responded to NO_3_^−^ supply. Together with the findings of GAs modulating the NO_3_^−^ uptake, GAs could regulate the transcript expression of *ZmNRT2.1* and *ZmNRT2.2* in maize in response to N supply, and might also be involved in modulating the iHATS-mediated NO_3_^−^ uptake, which will be further investigated.

The GAs also positively regulate other nutrient-acquisition related genes, such as the Pi transporter genes *SlPT2* and *SlPT7* [12], the K transporter gene *AtHAK5* [13], and the iron-uptake regulated genes *AtIRT1* (iron-regulated transporter) and *AtFRO2* (iron-regulated ferric chelate reductase) [48]. Moreover, the DELLA proteins interact with the transcription factor *AtFIT* and *AtbHLH38/39* for modulating FIT-regulated iron-uptake regulated genes [48]. Furthermore, the transcription factor *OsGRF4* drives the expression of *OsNRT2.3* and *OsGS1.2* in spite of being suppressed by the DELLA protein SLR1 in rice [10]. In the present study, the RNA-seq analysis revealed the presence of 287 TF genes responding to GA in the maize roots under the LN and SN conditions (Table S6). Both the binding-site prediction and co-expression analyses suggested that the *ERF, MYB, WRKY, TCP, bZIP, LBD, Dof, HsF, HB,* and *GAGT* family TFs might govern the expression of nitrate transporter genes (Figure 8). In *Arabidopsis*, several TFs governing the *NRT2.1* and *NRT1.1* expression have been identified, including the transcription factor LBD family genes (*LBD37/38/39*), NLP family genes (*NLP7*), TCP family genes (*TCP20*), SBP-box family genes (*SPL9*), and bZIP family genes (*TGA1/4*) [30]. Among these TFs, the transcript expressions of *ZmTCP33 and ZmLBD24* were significantly repressed in the *zmga3ox* plant under the LN and SN conditions (Figure 9a,b). The DELLA proteins could interact with class I TCP factors and block the TCP function by binding to their DNA-recognition domains in *Arabidopsis* [49]. However, the transcript expression of *ZmbZIP160* was upregulated in the *zmga3ox* plant (Figure 9c). More interestingly, the number of *MYB, WRKY,* and *ERF* family binding sites was enriched to the greatest extent, and the co-expression analysis indicated that *ZmNRT2.1* and *ZmNRT2.2* co-expressed with *ZmMYB14/24/37/48, ZmEREB93, and ZmWRKY22.* This aspect needs further exploration; therefore, subsequent research would be conducted to explore the transcriptional regulation of GAs modulating the expression of *ZmNRT2.1* or *ZmNRT1.1* in N uptake of maize.

## 4. Materials and Methods

### 4.1. Plant Materials, Growth Conditions, and Treatment

The CRISPR/Cas9 vector used to produce the z*mga3ox* (GRMZM2G036340) was obtained from the Maize Functional Genomic Project of China Agricultural University. The lines were produced in the maize (*Zea mays* L.) inbred line B73-329 background. For hydroponic experiments, the seeds were surface-sterilized in a 10% (*v/v*) H_2_O_2_ solution for 20 min and washed 5 times with distilled water. After that, the seeds germinated on the sand in a growth chamber, at 28/22 °C with a 16/8 h light/dark cycle, relative humidity controlled to approximately 70–80%. After germinated on the sand for 7 d, the uniform seedlings with two visible leaves were transferred to a hydroponic box (12 seedlings per pot) containing 5 L nutrient solution after the endosperm of each seedling was removed. The seedlings were supplied with half-strength complete nutrient solution for 2 d and then transferred to the full-strength culture solution supplied with different NO_3_^−^ concentrations (2.0 mM NO_3_^−^, SN; 0.05 mM NO_3_^−^, LN). To exclude the possibility of potassium (K^+^) interference, the concentrations of K^+^ in the LN solution were supplemented to the same levels as those of the SN solution using KCl. The complete nutrient solution for maize was modified Hoagland solution [50], consisting of 0.5 mM MgSO_4,_ 0.1 mM KH_2_PO_4_, 1 mM CaCl_2_, 0.1 mM EDTA-Fe, 2 mM KNO_3,_ and micronutrients (0.03 mM H_3_BO_3_, 0.0025 mM ZnSO_4_, 0.008 mM CuSO_4_, 0.005 mM MnSO_4_, and 0.0003 mM (NH4)_6_Mo_7_O_24_, and pH 5.8. The nutrient solution was renewed every other day. In addition, the nutrient solution, with or without 1 μM GA_3_ or 2 μM Ucz, was treated simultaneously with LN or SN treatment for 5 d.

For soil culture experiments, the wide-type and *zmga3ox* seeds were sown in plastic containers (20 × 20 × 30 cm deep) with a mixture of vermiculite and commercial garden soil (1:1; *v/v*) in a glasshouse. And 0.1 mM GA_3_ with 0.01% (*v/v*) Tween 20 were applied by foliar spray by using perfume bottles every other day.

### 4.2. The Measurement of Net NO_3_^−^ Flux, ^15^NO_3_^−^ Uptake and Total N Content

The net NO_3_^−^ fluxes were measured in the maturation zone (approximately 700 μm from the root tip) of PR using the non-invasive micro-test (NMT) system (Younger; Xuyue (Beijing) Sci & Tech) [51]. For each experimental group, maize seedlings were selected at 5 d after LN or SN treatment, and the roots were immediately equilibrated in a Petri dish containing 10 mL of measuring solution (0.1 mM NH_4_NO_3_, 0.1 mM KCl, 0.1 mM CaCl_2_, 0.3 mM MES, pH 6.0) for 20 min, and then transferred to another Petri dish containing fresh measuring solution. The NO_3_^−^ ion gradients close to the root surface (ca. 5 μm above the root surface) were determined by moving the flux microsensor between two positions (30 μm in distance) in the direction perpendicular to the root axis. The recording rate of ion flux was one reading per 6 s, and each measurement point was monitored for 10 min.

The N uptake assay in roots of the *zmga3ox* seedling was performed as described previously [10]. After LN or SN treatment for 5 d, the uniform seedlings were selected and rinsed the roots with 0.1 mM CaSO_4_ solution for 1 min, and then incubated in the nutrient solution containing 0.05 or 2 mM K^15^NO_3_ with a 99% atom excess of ^15^N, respectively, for 10min. After that, washed for 1 min in 0.1 mM CaSO_4_ solution before sampling. The samples were harvested and dried at 120 °C for 30 min, and then 65 °C for 72 h before being ground. The power was used for total ^15^N determination by isotope ratio mass spectrometry (Vario PYRO cube ISOprime 100, Cheadle Hulme, UK).

The roots and shoots of maize seedlings at 5 d after LN or SN treatment were separated for assay of N concentration following the Kjeldahl method [52]. Plant total N content was calculated as the product of N concentration and corresponding dry weight.

### 4.3. RNA Isolation and Reverse Transcription Quantitative Polymerase Chain Reaction (RT-qPCR) Analysis

Total RNA was isolated from each sample using the Plant RNeasy Mini kit (Tiangen, Beijing, China). Then the full-length cDNA was synthesized from 2 ug of RNA using Oligo d (T) primer and M-MLV reverse transcriptase (Takara, kusatsu, Japan). Quantitive PCR was conducted in an Applied Biosystems 7500 Fast Real-Time PCR System (Applied Biosystems, CA, USA) using SYBR^®^ Premix Ex Taq™ (Takara, Japan) following the manufacturer’s instructions. *ZmUBC* (ubiquitin C) was used to normalize gene expression [53]. Fold change of gene expression values were calculated using the 2^−ΔΔ*C*t^ method [54]. The primers for qRT–PCR are listed in Table S8.

### 4.4. RNA-Seq Analysis

Three biological RNA replicates were obtained from the root of wild-type and *zmga30x* plants at 12, 60, and 108 h after LN or SN treatment, each biological replicate contained roots from three plants. Library construction for transcriptome sequencing was performed using the NEBNext UltraTM RNA Library Prep Kit for Illumina (NEB, Ipswich, MA, USA). The clustering of the index-coded samples was performed on a cBot Cluster Generation System using a TruSeq PE Cluster Kit v4-cBot-HS (Illumia) according to the manufacturer’s instructions. After cluster generation, the library preparations were sequenced on an Illumina Hiseq 4000 platform, and paired-end 150 bp reads were generated. Clean data (clean reads) were obtained by removing reads with adapter reads containing ploy-N and low quality reads from raw data. At the same time, Q20, Q30, GC-content, and sequence duplication levels of the clean data were calculated. All the downstream analyses were based on clean data with high quality. The filtered reads from each sample were aligned to the maize reference genome (B73 RefGen_v3, http://www.maizegdb.org/assembly/) using TopHat2 [55]. Gene expression levels were estimated by fragments per kilobase of transcript per million reads (FPKM) to compare among different samples. EdgeR software package [56] was used to detect genes differentially expressed between wild-type and *zmga3ox* mutant. K-means clustering analysis was conducted by cluster 3.0 between LN and SN at three time points and visualized by using Java TreeView. A gene was regarded as significantly differentially expressed if the false discovery rate (FDR) adjusted to a *p*-value < 0.05 and log_2_ (fold change) ≥1.

Gene Ontology (GO) and Kyoto Encyclopedia of Genes and Genomes (KEGG) pathway annotation and enrichment analyses of DEGs were performed by the online agriGO software [57] and KOBAS 3.0 software [58], respectively.

### 4.5. Weighted Gene co-Expression Network Analysis (WGCNA)

The FPKM of DEGs was adopted to process the raw files as the input file for WGCNA. The expression data from all three time points were analyzed combined. The co-expression networks were constructed with R package WGCNA (version 1.66) [59]. The soft thresholding power was 14, minModuleSize was 20, deepSplit was 2, networkType was signed, and others were default settings following previous descriptions [60]. The co-expression network was visualized with Cytoscape 3.7.1 [61].

### 4.6. Transcription Factor-binding Site Prediction

Putative promoter sequences (2 kb upstream from the transcriptional start site) of DEGS were downloaded from Ensemble Plants (http://plants.ensembl.org). Then the TF-binding site prediction was performed with online software PlantRegMap (http://plantregmap.cbi.pku.edu.cn), according to Jin et al. [62].

### 4.7. GAs Concentration Analysis

The roots or shoots of wild-type and *zmga3ox* seedlings were collected at 5 d after LN or SN treatment. The endogenous concentration of GAs was determine by HPLC–MS/MS. Briefly, approximately 500 mg of tissue was ground in liquid nitrogen and then extracted with 5 mL of 90% aqueous MeOH. Simultaneously, 2 ng of each D-labeled gibberellin compound was added to each sample. The subsequent operation was performed as described by [63].

### 4.8. Assay of Physiological and Biochemical Properties

After LN treatment for 5 d, 0.2 g fresh leaves or roots of wild-type and *zmga3ox* seedlings were collected for analyzing the activities of GS and NR. Chlorophyll content was measured following Wu et al. [64]. The anthocyanin content was determined, as described previously [65]. The activities of GS and NR were determined, respectively, with the Glutamine Synthetase and Nitrate Reductase Kit (Solarbio LIFE SCIENCES, BC0910/ BC0080, Beijing, China) following the manufacturer’s instructions. The O_2_^−^ production rate was determined with the protocol described by Chen et al. [66]. The photosynthetic rate was assessed by using an LI-6400 XT portable photosynthetic system (LI-COR, Lincoln, NE, USA). Leaf area was calculated by using a Li-3000C portable leaf meter.

### 4.9. Statistical Analysis

The data were statistically analyzed using SAS 9.0 (SAS Institute Inc., Cary, NC, USA). The Student’s t-test was used for comparisons between two groups of data. For the data sets of more than two groups, one-way ANOVA with LSD (*p* < 0.05) was used.

## 5. Conclusions

Taken together, the results of the present study indicate that GA plays a significant role in the regulation of nitrogen uptake in the maize plant in response to N supply. LN significantly repressed the level of the bioactive GAs in the roots. Moreover, the shoots of the *zmga3ox* plants showed more sensitivity toward the LN stress, corresponding to the contribution of anthocyanin accumulation and the decrease of chlorophyll content. Also, the *zmga3ox* plants had low net NO_3_^−^ fluxes leading to lesser ^15^N content compared to the wild-type plants. The transcript expression of *ZmNRTs* was downregulated in the *zmga3ox* roots under the LN and SN conditions. These results suggested that GAs regulated N uptake involved in transcriptional regulation of *NRTs* expression and the physiological responses in maize responding to nitrogen availability. This research thus provides a valuable theoretical basis for improving N efficiency in crop production.

## Supplementary Materials

Supplementary materials can be found at https://www.mdpi.com/1422-0067/21/5/1824/s1.

## Abbreviations

GA	Gibberellin
GS	Glutamine Synthase
NR	Nitrate Reductase
LN	Low Nitrate
NUE	Nitrogen Use Efficiency
NMT	Non-Invasive Micro-Test System
NRT	Nitrate Transport
PR	Primary Root
SN	Sufficient Nitrate
Ucz	Uniconazole
FPKM	Fragments per Kilobase of Transcript per Million Reads
FDR	False Discovery Rate
DEGs	Differentially Expressed Genes
NRT	Nitrate Transporter
GRF4	Growth-Regulattion Factor 4
HAK5	High-Affinity Potassium Transporter 5;
SLR1	Slender Rice1
SlPT2	Tomato Phosphate Transporter
IRT1	Iron-Regulated Transporter 1
FRO2	Iron Regulated Ferric Chelate Reductase
WGCNA	Weighted Gene co-Expression Network Analysis
GO	Gene Ontology
KEGG	Kyoto Encyclopedia of Genes and Genomes
NPF	Nitrate Transporter1/Peptide Transporter Family
