# Supplementary material for: The Role of Gibberellins in Regulation of Nitrogen Uptake and Physiological Traits in Maize Responding to Nitrogen Availability
Source: Int J Mol Sci. 2020 Mar 6;21(5):1824. doi: 10.3390/ijms21051824 (PMC7084584; doi:10.3390/ijms21051824)
Supplement: Supplementary file 1 [file ijms-21-01824-s001.zip › Supporting figures and tables/Supporting Figures1-7.docx]

**Supporting information**

**Figure S1** The expression level of *ZmGA3ox* in the wild-type and zmga3ox mutant roots (a), and shoots (b).

**Figure S2** The photosynthetic rate, leaf area and superoxide anion radicals (O_2_•^-^) production rate in the wild-type and *zmga3ox* leaves under SN with or without GA_3_ application.

**Figure S3** The GA content and the expression levels of GA biosynthesis- and catabolic related genes in *zmga3ox* mutant seedlings responded to LN and SN.

**Figure S4** Plant growth and nitrogen accumulation properties in the wild-type and *zmga3ox* plants under LN and SN conditions.

**Figure S5** Clustering analysis DEGs expression pattern at 12h, 60h, and 108h after treatment.

**Figure S6** The expression levels of *ZmGOGAT1* (a), *ZmNiR1* (b), *ZmGln1* (c) and *ZmNIA1* (d) in wild-type and zmga3ox roots at different time points after LN or SN treatment.

**Figure S7** The changes of the activities of nitrate reductase (NR, a) and glutamine synthase (GS, b) in the wild-type and zmga3ox roots under LN and SN conditions.

Figure. S1. The expression level of *ZmGA3ox* in the wild-type and *zmga3ox* mutant roots (a), and shoots (b).

Figure S2. The photosynthetic rate, leaf area and superoxide anion radicals (O_2_•^-^) production rate in the wild-type and *zmga3ox* leaves under SN with or without GA_3_ application. (a) The photosynthetic rate in the wild-type and *zmga3ox* leaves at 5d after SN treated with or without GA_3_. (b) The leaf area in the wild-type and *zmga3ox* leaves at 5d after SN treated with or without GA_3_. (c) O_2_•^-^ production rate of in the wild-type and *zmga3ox* leaves at 5d after SN treated with or without GA_3_. Values were the means ± SD (n = 3). Different letters indicated significant difference calculated by Fisher’s LSD (*P* < 0.05).

Figure S3. The GA content and the expression levels of GA biosynthesis- and catabolic related genes in *zmga3ox* mutant seedlings responded to LN and SN. (a) The content of GA_3_ in the *zmga3ox* mutant roots at 5 d after LN and SN. Values were the means ± SD (n = 3). (b-d) The transcriptional levels of ent-kaurene synthases (b), GA 20-oxidases (c) and GA 2-oxidase (d) genes in the wild-type roots at 3 d after LN and SN. (b-d) Values were the means ± SD (n = 3). The asterisks indicated significant difference compared with the control as evaluated by student's t tests **p < 0.01.

Figure S4. Plant growth and nitrogen accumulation properties in the wild-type and *zmga3ox* plants under LN and SN conditions. (a-b) The dynamics changes of the shoot (a) and root (b) dry weight of wild-type and zmga3ox plants at 0, 1, 3, 5 and 7d after LN and SN treatment. (c-d) The inhibition ratio of LN to SN in the shoot (c) and root (c) dry weight of wild-type and *zmga3ox* plants. Values were the means ± SD (n = 6). (e-f) The dynamics changes of total nitrogen content in the shoot (e) and root (f) of the wild-type and *zmga3ox* plants at 0, 1, 3, 5 and 7d after LN or SN treatment. (g-h) The inhibition ratio of LN to SN in the total nitrogen content of the shoot (g) and root (h) of the wild-type and *zmga3ox* plants. Values were the means ± SD (n = 3). Different letters indicated significant difference between wild-type and zmga3ox plants at the same time points calculated by student's t tests (*P* < 0.05).

Figure S5. Heat map of of DEGs expression pattern at 12h, 60h, and 108h after treatment. Different expression levels are shown as log_2_FC, blue for down-regulation and red for up-regulation as shown in color bar.

Figure S6. The expression levels of *ZmGOGAT1* (a), *ZmNiR1* (b), *ZmGln1* (c) and *ZmNIA1* (d) in wild-type and *zmga3ox* roots at different time points after LN or SN treatment. Roots were harvested at 12, 60 and 108 h after LN or SN treatment. Values were the means ± SD (n =3). Different letters indicated significant difference between the wild-type and *zmga3ox* plants at the same time point calculated by Fisher‘s LSD (P < 0.05).

Figure S7. The changes of the activities of nitrate reductase (NR, a) and glutamine synthase (GS, b) in the wild-type and *zmga3ox* roots under LN and SN conditions. Values were the means ± SD (n = 3). Different letters indicated significant difference calculated by Fisher‘s LSD (P < 0.05).
